# Functional Mobility and Balance Confidence Measures Are Associated with Disability among Community-Dwelling Older Adults

**DOI:** 10.3390/medicina60091549

**Published:** 2024-09-22

**Authors:** Norah A. Alhwoaimel, Mohammed M. Alshehri, Ahmed S. Alhowimel, Aqeel M. Alenazi, Bader A. Alqahtani

**Affiliations:** 1 Department of Health and Rehabilitation Sciences, Prince Sattam Bin Abdulaziz University, Al-Kharj 11942, Saudi Arabiaaqeel.alanazi@psau.edu.sa (A.M.A.); 2 Department of Physical Therapy, Jazan University, Jazan 45142, Saudi Arabia

**Keywords:** mobility, balance confidence, disability, impairment, functional limitation, elderly, Saudi

## Abstract

*Background and Objectives:* Our objectives were to examine the association between balance confidence, functional mobility measures, and the presence of disability in older adults in Saudi Arabia and to identify the optimal cutoff scores for these measures that predict disability. *Materials and Methods:* A cross-sectional study was conducted among 324 community-dwelling older adults (65 ± 7 years, 59% women). Disability was assessed using the Arabic version of the basic activities of daily living (ADLs) scale. Balance confidence was evaluated using the Arabic version of the Activities -Specific Balance Confidence (ABC) scale, and functional mobility was assessed using the 30-Second Chair Stand Test (30s-CST). *Results:* The prevalence of disability was 33.6% among the participants. Logistic regression revealed a 6% increase in disability odds for each one-unit decrease in the 30s-CST (OR: 0.94) and a 3% increase per one-point decrease in the ABC score (OR: 0.97). The optimal cutoff scores were ≤5 repetitions for the 30s-CST (sensitivity: 74.4%; specificity: 51.4%; AUC: 0.64) and ≤40 for the ABC scale (sensitivity: 80.8%; specificity: 61.4%; AUC: 0.75). *Conclusions:* Impaired balance and functional mobility are significantly associated with disability among older adults. The 30s-CST and the ABC scale can be used as effective screening tools, but the cross-sectional design of the current study limits its generalizability. Longitudinal research is needed to validate these findings.

## 1. Introduction

In 2019, the global population aged 65 years and older was approximately 703 million, with projections forecasting a substantial increase to 1.5 billion by 2050 [1]. The Kingdom of Saudi Arabia (KSA) is undergoing a significant demographic transition marked by a rapidly aging population. By 2050, the proportion of older adults in Saudi Arabia is anticipated to rise from 9.5% in 2035 to 18% of the total population [2]. This demographic shift is expected to place considerable strain on the healthcare system due to an anticipated rise in age-related health issues and disabilities.

Aging is a dynamic, lifelong process characterized by progressive and cumulative physiological impairments that can impact the functional and motor capacities of older adults [3]. The World Health Organization (WHO) defines healthy aging as “the process of developing and maintaining the functional ability that enables well-being in older age” [4]. In its baseline report for the Decade of Healthy Ageing 2021–2030, the WHO underscored the importance of mobility as a crucial element of functional ability for older adults, irrespective of income levels [4]. Mobility plays a fundamental role in healthy aging, significantly affecting both physical and psychological well-being.

Mobility impairment, as measured by walking speed and balance performance, has been found to increase the risk of dependency in activities of daily living (ADLs) by 12 times in older adults aged ≥60 years [5]. An individual is classified as disabled when they exhibit partial or complete dependence in ADLs. Recent meta-analyses have indicated that muscle strength and mobility, as measured by gait speed, the Timed Up and Go Test, and the Chair Rise Test, are independent predictors of future dependency in both ADLs and instrumental activities of daily living (IADLs) [6]. The WHO’s report for the Decade of Healthy Ageing 2021–2030 also highlights that mobility in older adults is influenced by environmental factors and personal attitudes [4]. Perceptions of mobility barriers—such as fear of falling—or beliefs that physical activity increases the risk of injury can lead older adults to restrict their movements, further impacting their overall well-being [4,7].

Fear of falling can diminish older adults’ confidence and willingness to engage in daily activities, leading to further deterioration in mobility, balance, muscle strength, and overall quality of life [8,9]. Research by Portegijs et al. has demonstrated that reduced balance confidence and heightened fear of falling significantly impair mobility and overall functioning in older adults [10]. Balance confidence, defined as an individual’s belief in their ability to maintain balance, can exacerbate activity avoidance when it is low [11]. Decreased balance confidence, as indicated by an Activity-Specific Balance Confidence (ABC) scale score of less than 50, is associated with increased physical difficulties and reduced quality of life [12]. Recent studies have also established that balance confidence is a significant independent predictor of falls, with higher levels of balance confidence being linked to a considerably lower likelihood of experiencing falls [13].

In Saudi Arabia, recent research has highlighted a significant increase in fall-related incidents and disabilities among older adults over a 29-year period [14,15]. The incidence and prevalence of falls have risen for both genders, with males exhibiting a higher relative increase in prevalence rates (57% for males vs. 26% for females) [15]. Falls and associated disabilities are emerging as critical public health issues, especially given the growing aging population, necessitating targeted interventions. However, there is limited research on the association between disability, modifiable risk factors of falls such as mobility impairment, and balance confidence limitation in community-dwelling older adults. Therefore, it is crucial to study the relation between mobility disability and diminished balance confidence with disability in this population to determine risk factors, establish priorities for healthcare, and develop preventive and therapeutic strategies for these conditions. Hence, this study aimed to (1) examine the association between balance confidence, mobility measures as assessed by the ABC scale, and the 30-second sit-to-stand test, and the presence of any kind of disability as measured by the Arabic version of the ADL scale and (2) determine the optimal cutoff scores for balance confidence and mobility measures associated with any kind of disability using the Arabic version of the ADL scale.

## 2. Methods

### 2.1. Study Design and Participants

A cross-sectional correlation study was conducted to evaluate the association between balance, mobility, and having any kind of disability among older adults. Data were collected from the older adult community who lived in Saudi Arabia. The inclusion criteria included the age of 50 years and older, being able to read and write in the Arabic language, and being a Saudi citizen. The inclusion of a younger age group (50 years old) was considered due to the latest evidence that reported the prevalence rate of disability among adults aged less than 60 years old to be 3% [16]. Participants who were unable to read or write in Arabic and who were unable to provide informed consent were excluded.

### 2.2. Sample Size Calculation

A sample was calculated based on past evidence related to the prevalence of disability in Saudi Arabia [16]. The reported prevalence of disability was 3.3%. Therefore, we calculated the sample size using the formula [n = Z^2^ P(1 − P)/d^2^], where n = the sample size, Z = the Z-score statistic for a specific level of confidence (1.96), *p* = the prevalence of disability (3.3%), and d = the degree of precision (0.05). The sample size was estimated to be 318. To reach the size of the calculated sample, convenience sampling was used to recruit participants for this study.

### 2.3. Demographics and Clinical Variables

The data collection was conducted in-person by an independent trained physiotherapy researcher, starting with demographic and clinical data, including age, gender, body mass index (BMI), and chronic diseases. Major chronic diseases were collected via self-reported diagnoses, including hypertension, diabetes, cardiovascular disease, lung disease, neurological diseases, cancer, and arthritis.

### 2.4. Outcome Measures

Disability was measured using an Arabic version the of basic ADL scale [17]. The ADL scale is a 6-item scale that assesses overall functional activity in (1) bathing, (2) dressing, (3) going to the toilet, (4) transferring (movement), (5) continence, and (6) feeding. The Arabic version of the ADL scale has a good validity and reliability level, as it has been established in an Arabic elderly population living in nursing homes [17]. The Arabic ADL scale has excellent reliability, represented by a strong Cronbach alpha of 0.90, for the first three subscales on the ADL (bathing, dressing, and going to the toilet). The other three items (transferring, continence, and feeding) have acceptable levels of reliability, with a Cronbach alpha of 0.65 [17]. The six components of the ADL scale are scored 0, 0.5, or 1. The total ADL score ranges from 0 to 6, where 0 indicates being very dependent, 1–5 points indicates being partially dependent and 6 entails complete independence. Another calculation is performed, according to the developer, to convert the scores into 100s by adding the scores of the 6 items and dividing by 6. Then, the results are multiplied by 100 to convert them [17,18]. For the current study, any participant with a score of less than 100 was considered as having a disability [19,20]. This approach has been used in previous studies from the community. 

The 30-Second Chair Stand Test (30s-CST) was used to test functional mobility. The 30s-CST has an excellent test–retest reliability of 0.89 and a validity of 0.87 [21]. It is performed by asking a participant to complete as many full stands as possible from a chair without arms within 30 s. Based on this test, the physical performance of patients can be classified as low (30s-CST ≤ 8) or high (30s-CST > 8) [22].

Balance confidence was evaluated using the Arabic version of the Activity-Specific Balance Confidence (ABC) scale [11,23]. It is a valid and reliable self-rated questionnaire that consists of 16 items to assess a broad range of ADL confidence during ambulatory activities [11]. Respondents were asked to score their level of confidence in performing specific activities such as “picking up slipper from floor” and “walking in crowded mall”. Subjects rated themselves on a scale from 0 to 100, with 0% being no confidence and 100% being full confidence in the ability to perform the activity without losing balance. The total ABC scale score is calculated as the mean of the 16 responses. An ABC score below 67% is correlated with balance impairment and high fall risk in the elderly [24]. According to Powell and Myers, a score of >80% denotes excellent functioning, a score of 50% to 80% denotes moderate functioning, and a score of 50% denotes low functioning [11].

### 2.5. Statistical Analysis

Descriptive statistics were expressed as counts and percentages for categorical variables and means with standard deviation for continuous variables. Participants were classified into two categories according to the results of the Arabic version of the basic ADL scale (one group with no disability and other group with disability). Disability. Age, sex, body mass index (BMI), number of chronic conditions, and the 30-Second Chair Stand and ABC tests were recorded for each participant. The Chi square was used for comparing categorical variables between the groups with disability and no disability, and an independent t-test was used to compare the continuous variables.

To examine the association between balance and mobility measures with disability using the ADL scale, generalized linear models with binary logistic regression were used. Odds ratios (ORs) were calculated with the associated 95% confidence interval (95% CI) for the 30-Second Chair Stand and ABC tests. The model was adjusted for age, sex, multiple long-term conditions, and BMI category. 

To determine the cutoff scores for the balance and mobility measures associated with disability, a receiver operating characteristic (ROC) curve was utilized. The area under the ROC curve (AUC) indicates the overall accuracy of the model in detecting the presence or absence of disability among participants [25]. The AUC values were interpreted using an established guideline that classifies discriminant ability as follows: fail (0.5 ≤ AUC < 0.6), poor (0.6 ≤ AUC < 0.7), fair (0.7 ≤ AUC < 0.8), considerable (0.8 ≤ AUC < 0.9), and excellent (AUC > 0.9). To determine the best cutoff score, the Youden index (sensitivity + [1 − specificity]) was calculated, wherein the largest score was used as the cutoff. Sensitivity and specificity were calculated, indicating true positive and true negative results, respectively. An alpha level of 0.05 was used for all analyses. All analyses were performed using IBM SPSS for Macintosh (version 25.0; SPSS Inc., Chicago, IL, USA).

## 3. Results

### 3.1. Participant Characteristics

A total of 324 participants were included in the current analysis. The prevalence of disability was 33.6% among the study participants. The demographic and clinical characteristics of the participants are summarized in Table 1. Significant differences were observed between those with and without disability in terms of age, sex, BMI, MLTCs, performance on the 30s-CST, and total ABC test scores. The participants with disabilities were notably older, had higher BMIs, were more likely to have MLTCs, performed worse on the 30-second sit-to-stand test, and exhibited lower balance confidence compared with those without disabilities.

### 3.2. Association between Balance and Mobility Measures and Disability

The results of the binary logistic regression analysis examining the association between balance and mobility measures and disability are presented in Table 2. The regression model has been adjusted for potential confounding factors, including age, sex, MLTCs, and BMI.

The binary logistic regression results demonstrate a significant association (*p* = 0.046) between decreased mobility, as measured by the 30s-CST, and the presence of any kind of disability. Specifically, for every one-unit decrease in the 30s-CST score, there is a 6% increase in the odds of having a disability (OR: 0.94; 95% CI [0.88, 0.99]). Similarly, a decrease in balance confidence was significantly associated with disability (association (*p* = 0.002), with a 3% increase in the odds of having disability for every one-point decrease in the ABC score (OR: 0.97; 95% CI [0.95, 0.99]). These findings suggest that lower performance in both mobility and balance is associated with an increased likelihood of any kind of disability.

### 3.3. Determining Optimal Cutoff Scores

**Figure 1:**
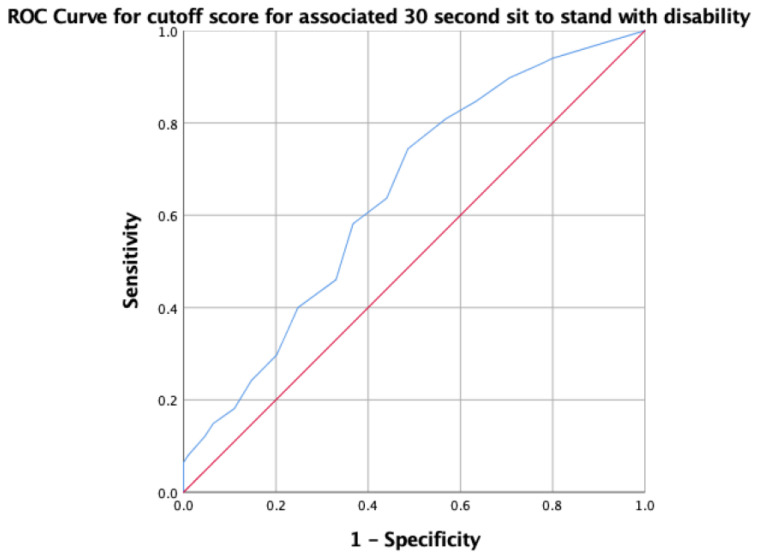
ROC curve for optimal cutoff score for 30s-CST associated with disability. The red line is the no discrimination line (AUC =0.5), and the blue line represents test with very high sensitivity (74.44%) but low specificity (51.4%).

The results for the optimal cutoff scores of the balance and mobility measures associated with disability are summarized in Table 3. For the 30-second sit-to-stand test, a cutoff score of ≤5 repetitions was identified, with a sensitivity of 74.44% and a specificity of 51.4%. This indicates that the individuals who performed fewer than five repetitions were more likely to have disability. The accuracy of this cutoff score is illustrated by the ROC curve in Figure 1, showing an AUC of 0.64 for the 30-second sit-to-stand test, reflecting poor discriminant ability.

**Figure 2:**
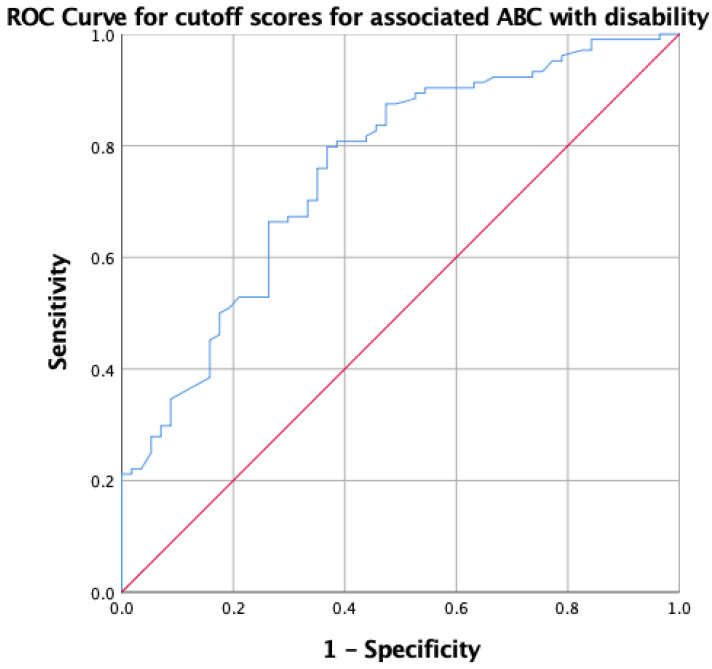
ROC curve for optimal cutoff scores for ABC associated with disability. The red line is the no discrimination line (AUC = 0.5), and the blue line represents test with very high sensitivity (80.8%) but low specificity (61.4%).

For the ABC scale, a cutoff score of ≤40 was found to be associated with disability, demonstrating a sensitivity of 80.8% and a specificity of 61.4%. The ROC curve for the ABC scale optimal score illustrated in Figure 2 shows an AUC of 0.75, indicating fair discriminatory ability.

## 4. Discussion

This study aimed to investigate the association between balance and mobility measures and having any kind of disability in performing ADLs among older adults in Saudi Arabia. The main findings from this study showed that reduction in mobility (measured by the 30s-CST) and balance confidence decline (measured by the ABC scale) were associated with any kind of disability among older adults. A score of ≤5 times sitting to standing on the 30s-CST and a score of ≤40 on the ABC scale were the best cutoff points associated with disability.

In our study, we found that participants with lower mobility scores, as measured by the 30-second sit-to-stand test, were more likely to have a disability. This finding aligns with previous research that has also demonstrated an association between reduced mobility and the presence of ADL disability in older adults [5,26,27]. A longitudinal study with a 10-year follow-up, of 3156 community-dwelling older adults, examined risk factors for mortality and physical disability. It found that low mobility, as measured by gait speed, was significantly inversely associated with the risk of developing a disability (*p* < 0.001) [27]. Similarly, a longitudinal study conducted in Sweden found that mobility limitations—defined as a one-leg balance stand of less than 5 s or a walking speed of less than 0.8 m/s—were associated with an odds ratio of 12.9 for incident disability over a six-year follow-up period, indicating a significant likelihood of developing ADL disability in individuals with limited mobility [5]. Furthermore, a recent meta-analysis that included 40 studies and 85,515 older adults further investigated the association between mobility capacity and incident ADL disability in community-dwelling older adults [28]. The results revealed that each second increase in the Timed Up and Go Test and Chair Rise Test performances corresponded to risk ratios of 1.15 and 1.07, respectively, for incidents of disability. These findings can be explained by changes in muscle mass, muscle strength, and muscle fat infiltration in older adults. A cohort study involving 3075 older adults (aged 70–79 years) reported that lower muscle mass (smaller cross-sectional thigh muscle area), reduced knee extensor muscle strength, and greater fat infiltration in the muscle were all associated with an increased risk of mobility loss (defined as difficulty of walking a quarter mile or climbing 10 steps) [29]. Overall, our findings, along with previous research, emphasize the critical need to incorporate standardized mobility assessments into routine health evaluations for older adults to effectively identify those at risk of disability.

However, several critical aspects must be considered when interpreting the mobility limitations in our study compared to previous studies [5,27,28,29]. The cutoff points used to define impairment for each mobility test differed between studies [5,27,28]. Our study used the 30-second sit-to-stand test, while others used the one-leg balance stand test and walking speed [5]; gait speed [27]; and a combination of gait speed, the Timed Up and Go Test, and the Chair Rise Test [28]. These different measurements of mobility have varying levels of difficulty, indicating the different capacities required to perform each test.

Our study also found a significant association between low balance confidence in daily activities and the prevalence of disability among older adults, with a cutoff score of ≤40 on the ABC scale being associated with disability. This finding is consistent with previous research that demonstrated that older adults aged 50 to 80 with ABC scores of below 50 experienced significantly higher levels of functional disability (*p* < 0.001) compared with those with scores of 50 or above [12]. The variation in cutoff scores between our study and previous findings can be attributed to the use of different outcome measures for disability, as the latter defined disability based on the WOMAC functional disability scale [12]. Additionally, their sample size was relatively small (n = 47). Furthermore, a two-year longitudinal study observed a 5% decline in ABC scale scores among community-dwelling older women, which was significantly associated with increased disability [30]. However, their definition of disability was different, as they measured it by the Physical Activity Scale for the Elderly (PASE) and the Survey of Activities and Fear of Falling in the Elderly [30]. Despite the differences in disability outcome measures between our study and previous research, these findings emphasize the importance of considering balance confidence as a key factor in identifying the risk of disability. This, in turn, can inform the design of targeted interventions aimed at reducing the risk of disability.

The decline in balance confidence among older adults may be attributed to factors such as multimorbidity, reduced balance ability, and decreased mobility [31,32,33,34]. Notably, balance performance accounts for 57% of the variance in balance confidence among community-dwelling older adults [32].

Our study established cutoff points for the 30s-CST and the ABC scale to predict the presence of ADL disability in older adults. The optimal cutoff scores identified were ≤5 repetitions for the 30s-CST and ≤40 for the ABC scale. Previous research has provided performance standards for the 30s-CST among men and women aged 60–94, indicating that completing 9 to 17 repetitions correlates with a fitness level that supports physical independence later in life [35]. While several studies have determined the cutoff scores for the ABC scale to predict the risk of falls and recurrent falls in the elderly population [24,36,37], there has been no previous research focused on identifying the optimal cutoff points for the 30s-CST and ABC scale specifically associated with ADL disability. This gap highlights the need for future research to predict functional disability among older adults.

Nevertheless, several limitations of this study should be acknowledged. First, the assessment of ADL disability relied on self-reported measures, which may have been subject to reporting bias. Second, the cross-sectional design of this study limited the ability to infer causality between balance, mobility, and the presence of disability.

## 5. Conclusions

This study demonstrates a significant association between impaired mobility and balance confidence and the presence of disability among older adults in Saudi Arabia. The 30-second sit-to-stand test demonstrated limited discriminatory ability, whereas the ABC scale had a fair discriminatory ability in distinguishing between older adults with and without disability. The moderate predictive power of the identified cutoff points suggests that these tools might be beneficial in clinical practice when used in conjunction with comprehensive clinical evaluations to assess the risk of disability in older adults. Identifying modifiable risk factors associated with disability enables practitioners to implement targeted interventions, such as exercise programs and fall prevention strategies, to potentially reduce the prevalence of disability. Additionally, these findings highlight the need for public health policies that support routine screening for balance and mobility impairments in both community and clinical settings.

Further research is required to establish causality through longitudinal studies that provide deeper insights into how balance confidence and mobility impairments influence disability trajectories over time in older adults. Additionally, future studies should integrate both subjective and objective measures of disability to ensure more comprehensive and accurate evaluation.

## Figures and Tables

**Table 1:** Participants’ demographics and clinical factors.

Factors	No Disability (n = 215)	With Disability (n = 109)	*p*-Value
Age (mean ± SD)	64.93 ± 7	68.19 ± 8	<0.001
Sex, female (% within disability status)	115 (53.5)	78 (71.6)	0.002
BMI (mean ± SD)	27.83 ± 4.84	30.28 ± 6.64	0.001
MLTCs (% within disability status)			0.004
No chronic disease	54 (25.1)	18 (16.5)	
One chronic disease	69 (32.1)	23 (21.1)	
MLTCs	92 (42.8)	68 (62.4)	
30-Second Chair Stand (mean ± SD)	8.55 ± 4.92	5.94 ± 4.67	<0.001
Total ABC test * (mean ± SD)	66.15 ± 26.50	38.82 ± 28.89	<0.001

* Due to missing variables for the total ABC test, the number of participants is different: no disability (n = 104) and disability (n = 57). SD: standard deviation. BMI: body mass index. MLTCs: multiple long-term conditions.

**Table 2:** Binary logistic regression for disability using the ADL scale versus mobility and balance measures.

	OR [95%CI]	*p*-Value
30-Second Chair Stand (n = 324)	0.94 [0.88, 0.99]	0.046
ABC (n = 161)	0.97 [0.95, 0.99]	0.002

OR: odds ratio, CI: confidence interval, ABC: Activity-Specific Balance Confidence scale. The model was adjusted for age, sex, MLTCs, and BMI.

**Table 3:** Cutoff scores for mobility and balance measures that are associated with disability.

Variables	AUC (95% CI)	Cutoff Score (Sensitivity, Specificity) *
30-Second Chair Stand (n = 324)	0.64 (0.58, 0.71)	<5.5 (74.44%, 51.4%)
ABC (n = 161)	0.75 (0.67, 0.83)	<40 (80.8%, 61.4%)

AUC: area under the curve, CI: confidence interval, ABC: Activity-Specific Balance Confidence scale. * Selected by highest Youden index.

## Data Availability

The datasets used and/or analyzed during the current study are available from the corresponding author upon reasonable request.
